# To GP or not to GP: a natural experiment in children triaged to see a GP in a tertiary paediatric emergency department (ED)

**DOI:** 10.1136/bmjqs-2017-006605

**Published:** 2017-09-29

**Authors:** Laurie Smith, Yajur Narang, Ana Belen Ibarz Pavon, Karl Edwardson, Simon Bowers, Katharine Jones, Steve Lane, Mary Ryan, David Taylor-Robinson, Enitan Carrol

**Affiliations:** 1 Institute of Infection and Global Health, University of Liverpool, Liverpool, UK; 2 Institute of Infection and Global Health, University of Liverpool, Liverpool, UK; 3 Alder Hey Children’s NHS Foundation Trust, Liverpool, UK; 4 Liverpool Clinical Commissioning Group, Liverpool, UK; 5 Urgent Care 24, Liverpool, UK; 6 Department of Biostatistics, University of Liverpool, Liverpool, UK; 7 Institute of Psychology, Health and Society, University of Liverpool, Liverpool, UK

**Keywords:** primary care, emergency department, quality improvement methodologies

## Abstract

**Objective:**

To evaluate the impact of integrating a general practitioner (GP) into a tertiary paediatric emergency department (ED) on admissions, waiting times and antibiotic prescriptions.

**Design:**

Retrospective cohort study.

**Setting:**

Alder Hey Children’s NHS Foundation Trust, a tertiary paediatric hospital in Liverpool, UK.

**Participants:**

From October 2014, a GP was colocated within the ED, from 14:00 to 22:00 hours, 7 days a week. Children triaged green on the Manchester Triage System without any comorbidities were classed as ‘GP appropriate’. The natural experiment compared patients triaged as ‘GP appropriate’ and able to be seen by a GP between 14:00 and 22:00 hours (GP group) to patients triaged as ‘GP appropriate’ seen outside of the hours when a GP was available (ED group). Intention-to-treat (ITT) analysis was used to assess the main outcomes.

**Results:**

5223 patients were designated as ‘GP appropriate’—18.2% of the total attendances to the ED over the study period. There were 2821 (54%) in the GP group and 2402 (46%) in the ED group. The median duration of stay in the ED was 94 min (IQR 63–141) for the GP group compared with 113 min (IQR 70–167) for the ED group (p<0.0005). Using the ITT analysis equivalent, we demonstrated that the GP group were less likely to: be admitted to hospital (2.2% vs 6.5%, OR 0.32, 95% CI 0.24 to 0.44), wait longer than 4 hours (2.3% vs 5.1%, OR 0.45, 95% CI 0.33 to 0.61) or leave before being seen (3.1% vs 5.7%, OR 0.53, 95% CI 0.41 to 0.70), but more likely to receive antibiotics (26.1% vs 20.5%, OR 1.37, 95% CI 1.10 to 1.56). Sensitivity analyses yielded similar results.

**Conclusions:**

Introducing a GP to a paediatric ED service can significantly reduce waiting times and admissions, but may lead to more antibiotic prescribing. This study demonstrates a novel, potentially more efficient ED care pathway in the current context of rising demand for children’s emergency services.

## Introduction

The total number of visits to emergency departments (ED) in the National Health Service (NHS) in England exceeded 22 million in 2014–2015, an increase of 35% over the last decade.^1^ Over 30% of these visits could potentially be managed in primary healthcare facilities.^2–8^ A number of strategies have been developed to manage demand on ED services in the UK (eg, telephone triage, walk-in centres and minor injury units), yet visits continue to increase. Perceived severity of the condition, reputation of the hospital, availability of diagnostic and treatment options, accessibility of primary care or out-of-hours services are common reasoning behind the decision to attend ED.^5 9 10^ Moreover, parents with young children are more likely to attend than those with older children due to increased concern over the potential severity of the condition and need for urgent treatment.^2 5–7 11–13^

A key challenge for paediatrics is to recognise seriously ill children. The most common medical presentations in ED are breathing difficulty, febrile illness, diarrhoea and vomiting, abdominal pain, seizures and rash, all of which could be potentially serious.^14^ While most cases eventually turn out to be symptoms of a minor illness that does not require emergency treatment,^11 13 15^ it is critical that seriously ill children are not missed. Long wait times and overcrowding in EDs are associated with delays in delivering urgent treatments such as antibiotics for sepsis.^16^ In recognition of this, one of the key recommendations of a report by the Royal College of Emergency Medicine was colocation of out-of-hours primary healthcare provision within ED.^17^ These services may deliver shorter waiting times, a reduction in additional diagnostic tests and examinations, and increased patient satisfaction, although their cost-effectiveness remains unclear.^18–23^ Evaluations have been conducted in general hospitals, but not in a paediatric setting. Our study makes use of a natural experiment by assessing the impact of the introduction of a primary care service on waiting times, admissions and antibiotic prescribing within a large paediatric ED in the UK.

## Methods

### Study setting, population and design

The study was conducted at Alder Hey Children’s NHS Foundation Trust, one of Europe’s largest tertiary paediatric hospitals with 270 beds located in Liverpool, England. The hospital serves a population of 1.3 million, and the ED receives 56 000 children annually. Children are triaged on arrival, and care is allocated based on clinical urgency. The department is staffed by at least one senior doctor from 08:00 to midnight, and by a combination of general practice, emergency and paediatric trainees along with emergency and advanced nurse practitioners over a 24-hour period.

From 1 October 2014 to 31 March 2015, a general practitioner (GP) employed by a Liverpool out-of-hours GP service (Urgent Care 24) was placed in the ED as a separate, but colocated service. The service had its own clinical and clerical staff, with a general practice-based electronic information system. The service ran between 14:00 and 22:00 hours, 7 days a week. The GPs were there almost all of the time, there may have been the very occasional day when they were not there, but the allocation only took place if the GP was present.

All patients were initially seen by a qualified ED nurse who conducted an initial evaluation using the Manchester Triage System (MTS) reference.^24^ The MTS was used to identify children who triaged ‘Green’. These are standard cases without immediate danger or distress, and identify a group of less urgent patients. The MTS assessments follow a flow chart based on the patient’s reason for contacting the ED. The chart begins by identifying possible criteria indicating life-threatening conditions for the patient, and if none of these conditions are present, the nurse continues along the flow chart asking questions until the nurse assigns the patient an appropriate category. The nurse’s experience can contribute to the assessment, but on the other hand, the risk of the nurse missing serious conditions is reduced because the flow chart forces the nurse to ask key questions and make vital inquiries.

Children who triaged MTS ‘Green’, without any complicating factors or comorbidities, and presenting with conditions that were deemed suitable to be treated by a GP were labelled ‘GP appropriate’. The GP could refuse to see the patients and have them re-enter the ED queue if they were unhappy with the allocation. Parents could also refuse to see the GP. In addition, nursing staff could use ‘common sense’ to allocate certain patients to ED if they deemed it appropriate. Children labelled as ‘GP appropriate’ outside the GP working hours of 14:00–22:00 were treated by the ED clinical staff following the standard procedures of the service. Patients seen by the GP and referred to the ED (n=178) were analysed within the GP allocated group.

The introduction of this service presented an opportunity to evaluate a ‘natural experiment’^25^ conducting a retrospective evaluation on the cohort of children attending the ED over a 6-month period between 1 October 2014 and 31 March 2015, triaged as ‘GP appropriate’. The main comparison was between patients triaged as ‘GP appropriate’ and seen by a GP between 14:00 and 22:00 hours (GP group) compared with patients triaged as ‘GP appropriate’ seen outside of those hours (ED group) when the GP service was not available. The GP group were analysed as the GP group regardless of whether they refused to see the GP or the GP refused to see them.

### Outcomes and covariates

Data were collected retrospectively on all patients over the period of the study from ED and GP service databases. For all cases, information on arrival and discharge date and time, reason for attending, initial diagnosis, final diagnosis, discharge status, prescription of antibiotic and attending physician (GP or ED doctor) were available. Additionally, information on age, gender and home postcode for each patient was also collected. Postcodes were used to generate IMD 2015 (Index of Multiple Deprivation 2015) scores for each patient. The following outcomes were considered: admission to hospital (yes/no), total time spent in the ED, exceeding the UK government’s 4-hour waiting target (yes/no), antibiotic prescription (yes/no) and leaving before being seen by a doctor (yes/no).

### Analysis strategy

First, descriptive statistics were generated for both groups. Differences in proportions were compared using the χ^2^ tests and differences in continuous measures assessed using the Mann-Whitney U test. We then used the equivalent of an intention-to-treat (ITT) analysis to compare the GP and ED groups to assess the primary outcomes. ORs were calculated using logistic regression to assess effect sizes between the two groups, along with 95% CIs, adjusting for age, deprivation score and diagnostic category. All statistical analyses were conducted using STATA V.12 (Stata, College Station, TX, USA) or SPSS V.20 (IBM Released 2013. IBM SPSS Statistics for Windows, V.22.0).

### Robustness/sensitivity analyses

We repeated our main analysis using the equivalent of a per-protocol (PP) analysis (comparing only those who actually were treated by the GP in GP hours to those seen by ED staff in ED hours); restricting the sample to those seen between 10:00 and 18:00 hours since daytime circumstances between the comparison groups are fairly similar in terms of children being awake, parents working, ED staffing and other such unmeasured confounders; and stratifying the analysis by age (under and over 5 years).

## Results

A total of 28 655 patients attended the ED at Alder Hey in the 6 months between 1 October 2014 and 31 March 2015, of which 5223 (18.2%) were triaged ‘GP appropriate’ and included in the study cohort. Of the ‘GP appropriate’ patients, 2821 (54%) were seen between 14:00 and 22:00 hours, and 2402 (46%) were seen outside these hours. Tables 1 and 2 show the characteristics and discharge status of both groups.

**Table 1:** Characteristics of the ‘GP appropriate’ patients attending the ED at Alder Hey

Variable	GP hours: 2821 (54%)	ED hours: 2402 (46%)	Total hours: 5223	Significance
Gender				p=0.88*
Female	1380 (48.9%)	1170 (48.7%)	2550 (48.8%)	
Male	1441 (51.1%)	1232 (51.3%)	2673 (51.2%)	
Age category				p<0.001*
<1 year	761 (21%)	574 (23.9%)	1335 (25.6%)	
1–5 years	1244 (44.1%)	1006 (41.9%)	2250 (43.1%)	
5–10 years	479 (17.0%)	481 (20.0%)	960 (18.4%)	
>10 years	337 (11.9%)	341 (14.2%)	678 (13.0%)	
Age (months)				p<0.001†
Median (IQR)	29 (10–70)	34 (12–80)	31 (11–75)	
Deprivation quintiles				p=0.27*‡
1	67 (2.4%)	76 (3.2%)	143 (2.7%)	
2	144 (5.1%)	129 (5.4%)	273 (5.2%)	
3	290 (10.3%)	238 (9.9%)	528 (10.1%)	
4	386 (13.7%)	308 (12.8%)	694 (13.3%)	
5	1933 (68.5%)	1647 (68.6%)	3180 (68.5%)	
Time of arrival (hours)				NA
02:00–05:59			111 (4.6%)	
06:00–09:59			545 (22.7%)	
10:00–13:59			1365 (56.8%)	
14:00–17:59	1416 (50.2%)			
18:00–21:59	1405 (49.8%)			
22:00–01:59			381 (15.9%)	
Diagnosis				NA§
Infection	1438 (51.0%)	1191 (49.6%)	2629 (50.3%)	
GI/abdominal	540 (19.1%)	449 (18.7%)	989 (18.9%)	
Dermatological	292 (10.4%)	193 (8.0%)	485 (9.3%)	
Minor injuries	248 (8.8%)	168 (7.0%)	416 (8.0%)	
Central nervous system	51 (1.8%)	46 (1.8%)	96 (1.8%)	
Allergy/immunology	47 (1.7%)	23 (1.0%)	70 (1.3%)	
ENT	15 (0.5%)	24(1.0%)	39 (0.7%)	
Cardiovascular	21 (0.7%)	14 (0.8%)	35 (0.7%)	
Ophthalmological	6 (0.2%)	27 (1.1%)	33 (0.6%)	
Respiratory	15 (0.5%)	16 (0.7%)	31 (0.6%)	
Nothing abnormal	12 (0.4%)	18 (0.7%)	30 (0.6%)	
Other	136 (4.8%)	234 (9.7%)	370 (7.1%)	

*χ^2^ test.

†Mann-Whitney U test.

‡NA=1 and 4, respectively.

§Too many categories.

ED, emergency department; ENT, ear-nose-throat; GI, gastrointestinal illness; GP, general practitioner; NA, not applicable.

**Table 2:** Discharge status (ITT analysis) of children in the GP group, the GP to ED group (initially assigned to the GP group but subsequently referred to the ED) and the ED group

Discharge	GP group	GP to ED group	ED group	Total
Own GP follow-up	1179 (43.8%)	19 (10.7%)	640 (27.2%)	1838 (35.2%)
Discharged	1364 (50.7%)	89 (50.0%)	1331 (56.5%)	2784 (53.3%)
Admitted	27 (1.0%)	51 (28.7%)	140 (5.9%)	218 (4.2%)
Outpatient services	23 (0.9%)	13 (7.3%)	89 (3.8%)	125 (2.4%)
A&E services	6 (0.2%)	2 (1.1%)	15 (0.6%)	23 (0.4%)
Community service	2 (0.1%)	2 (1.1%)	4 (0.2%)	8 (0.2%)
Dentist	1 (0.6%)	1 (0.1%)	2 (0.1%)	
Left before being seen	88 (3.3%)	1 (0.6%)	136 (5.8%)	225 (4.3%)

A&E, accident & emergency department services; ED, emergency department; GP, general practitioner; ITT, intention to treat.

Patient age ranged from 4 days to 16.6 years (median 31 months), and 48.8% were male. No significant differences were observed in sex between those attended by the GP and ED, but those seen by the GP were younger (median age 29 months) compared with those seen by the ED (median age 34 months). No significant differences were observed in the distribution of deprivation scores between the GP and ED groups. Over 80% of the ED patients arrived in hospital between 10:00 and 21:59 hours, and 111 patients (2.1%) did so in the early hours of the morning (02:00–05:59).

### Diagnostic category

The proportions of patients in each diagnostic category were similar in the GP group and the ED group (table 1 and online supplementary table S1). Infection diagnoses (eg, ear infection, urinary tract infection, conjunctivitis, balanitis) were recorded for 2629 (50.3%) patients. In the GP group, 1438 (51.0%) had infection diagnoses, compared with 1191 (49.6%) in the ED group. Gastrointestinal illness/abdominal pain was the second most frequent diagnostic category, with 989 cases (18.9%). The majority of these patients (540, 55%) were seen by the GP. Dermatological conditions and minor injuries (especially head injuries) were seen in 485 (9.3%) and 416 (8.0%) cases, respectively, and 60% of patients with either of these diagnoses were seen by the GP (table 1).

10.1136/bmjqs-2017-006605.supp1 Supplementary file 1

### Discharge status

During the evaluation period, 2821 (54%) patients were in the GP group. Of those, 2689 (95.3%) were managed by the GP alone, and 178 (6.3%) were referred to the ED for further evaluation. Fifty-one (28.7%) of the referred patients were hospitalised, and the two common diagnoses among these were infection (n=27, 52.9%) and gastrointestinal/abdominal conditions (n=12, 23.5%).

A total of 94.6% of children in the GP group (and seen only by the GP) were discharged with no further action, or advised to seek follow-up with their own GP. Twenty-seven children (1.0%) were admitted, and 31 (1.2%) were referred to other services (23 to outpatient services, 6 to further ED clinic, 2 to community services). Eighty-eight (3.3%) left before being treated. In comparison, of the 2356 in the ED group (excluding those initially seen by GP then referred to ED), 1971 (83.7%) were treated and discharged without further follow-up or discharged to their own GP for follow-up, 109 (4.6%) were referred to other services and 140 (5.9%) were admitted. One hundred and thirty-six (5.8%) left before being seen by the ED team (table 2 and online supplementary table S2).

### Evaluation of outcomes of interest for the intervention

#### Admission to hospital

In total, 78 (2.7%) patients in the GP group and 140 (5.9%) patients in the ED group were admitted. Adjusting for age, deprivation score and diagnostic category, the odds of being admitted were significantly lower (55%) if the patient was seen by the GP (OR 0.45; 95% CI 0.34 to 0.60; p<0.001) (table 3 and online supplementary table S3).

**Table 3:** ORs for the outcomes of admission, antibiotics, exceeding the 4-hour target and leaving before being seen by ITT analysis

Outcome	GP group	ED group	OR
Admitted	78 (2.7%)	140 (5.9%)	0.45 (0.34, 0.60)
Antibiotics	715 (26.0%)	462 (20.5%)	1.36 (1.19, 1.55)
Wait exceeded 4 hours	68 (2.4%)	120 (5.1%)	0.46 (0.34, 0.63)
Left before seen	89 (3.1%)	136 (5.7%)	0.53 (0.41, 0.70)

ED, emergency department; GP, general practitioner; ITT, intention to treat.

#### Antibiotic prescription

**Figure 1:**
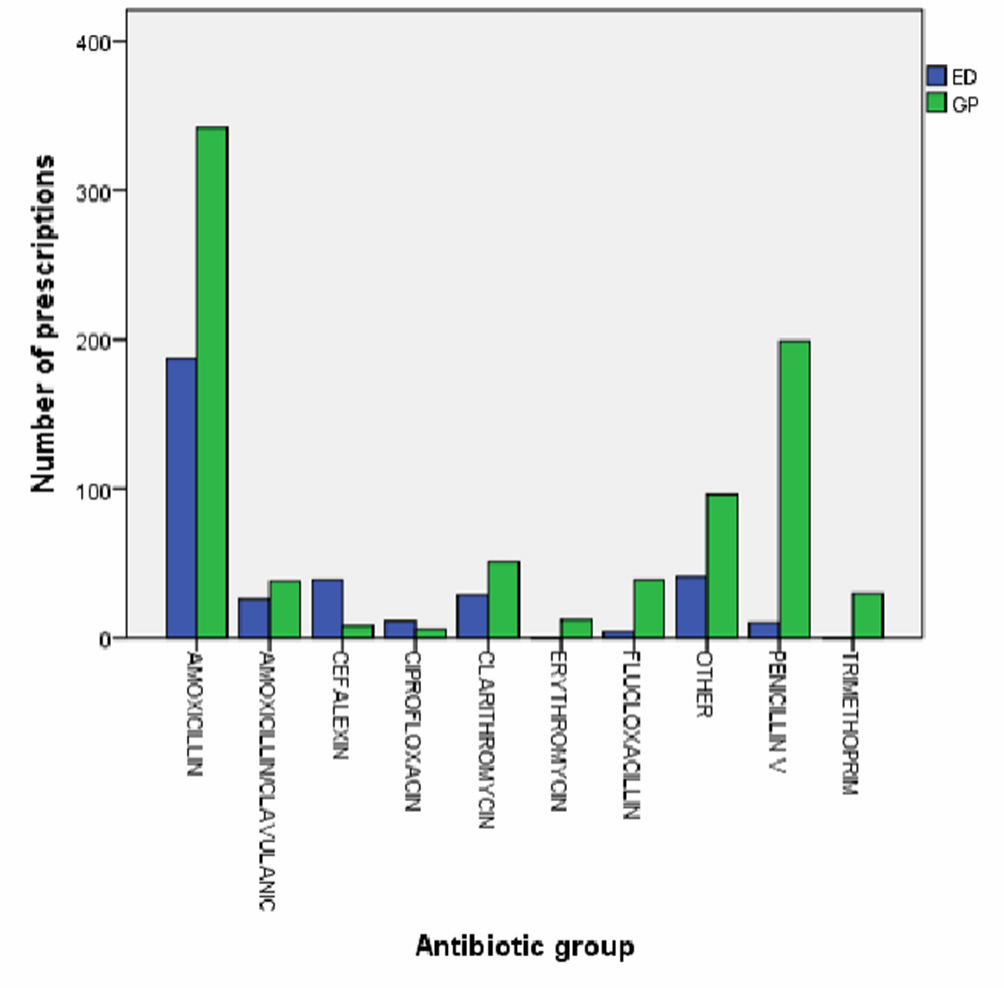
Antibiotics prescribed to ‘GP appropriate’ patients according to GP group and ED group by number of prescription. ED, emergency department; GP, general practitioner.

Antibiotics (oral and topical) were prescribed to 1177 patients. A total of 715 (26.0%) in the GP group and 462 (20.5%) in the ED group were prescribed antibiotics. The majority of antibiotic prescriptions were given to patients diagnosed with infection (987, 83.9%). Those treated by the GP were 36% more likely to receive antibiotics using ITT analysis (OR 1.36; 95% CI 1.19 to 1.55; p<0.001) (table 3 and online supplementary table S3). Amoxicillin was the most commonly prescribed antibiotic by both the GP and the ED clinicians. Penicillin V was prescribed significantly more frequently by the GP than the ED (figure 1 and online supplementary figure S1).

10.1136/bmjqs-2017-006605.supp2 Supplementary file 2

#### Exceeding the 4-hour government target

Overall, patients who attended the ED and were designated as ‘GP appropriate’ spent a median of 102 min from arrival to discharge, with times ranging from 14 min to 9 hours. The median duration of stay in the ED was 94 min (IQR 63–141) for the GP group compared with 113 min (IQR 70–167) for the ED group (p<0.005).

Allocation to the GP was associated with decreased odds of patients exceeding the 4-hour waiting time target set by the Department of Health^26^ by 54% (OR 0.46; 95% CI 0.33 to 0.63; p<0.001) for ITT analysis (table 3 and online supplementary table S3).

#### Leaving before being seen

Patients in the GP group were less likely to leave the department without being seen (OR 0.53; 95% CI 0.41 to 0.70; p<0.001) for ITT analysis (table 3 and online supplementary table S3).

#### Sensitivity analyses

Our results were similar using the equivalent of the PP analysis (online supplementary table S2 and S3). We conducted a sensitivity analysis of patients seen between 10:00 and 18:00 hours, comparing patients seen between 10:00 and 14:00 hours for the ED group and 14:00 and 18:00 hours for the GP group. This was to compare outcomes within a period when arguably factors potentially related to time of day do not vary. During the hours of 10:00–14:00, staffing levels and levels of services available in the hospital (phlebotomy, radiology, laboratory) are more homogeneous.

The conclusions are the same (online supplementary tables S4–S6). We also conducted a sensitivity analysis comparing age less than or equal to 5 years and age greater than 5 years, and demonstrated that there is no difference in outcomes between under 5-year-olds and over 5-year-olds (online supplementary table S7). This supports our finding that there is no age bias in the outcomes.

We assessed how big the unobserved confounding would need to be to change the estimated treatment effects significantly, and this provides further reassurance that our results are robust. We split the data by first half of each month versus the second half of each month and found no difference in outcomes (online supplementary table S8).

## Discussion

During a 6-month pilot scheme which colocated a primary care GP service in a busy paediatric ED, patients seen during the hours when the GP was available were significantly less likely to be admitted, exceed the 4-hour waiting target or leave before being seen, but more likely to receive antibiotics. The introduction of a new service within a tertiary paediatric ED presents the opportunity for a natural experiment, with the exposure group being seen by a GP. We have performed both ITT analysis (all those who were eligible to receive the new intervention rather than just for those who actually received it) and PP analysis (those who were eligible for the new treatment and actually received it). The conclusions remain the same with both analyses.

A report commissioned by the UK Department of Health and published by the Primary Care Foundation in 2009 evaluated different aspects of the integration of primary care services colocated within or alongside EDs in England.^20^ The report estimated that between 10% and 30% of cases attending EDs in England could be appropriately managed by primary care services. This is consistent with our finding of a little over 18% of cases attending the ED in Alder Hey were deemed as ‘GP appropriate’. Our intervention was implemented to try and address increased workload and long waiting times in the ED. We evaluated the first 6 months in order to produce evidence of the utility and value of the intervention. The service is currently undergoing continuous modification in order to best serve the needs of patients and derive most benefit from the primary care team, and further studies will evaluate these aspects, including health economic evaluation.

The UK Primary Care Foundation report highlighted that although the colocation of GP services within ED (adult EDs only) was operating in around two-thirds of the services in England, there was a lack of evidence on whether this model provided any benefits. The main drivers for the decision to introduce primary care within the ED appeared to be reducing economic costs and meeting the 4-hour target. Some analyses suggest colocation of GPs in ED is unlikely to have a significant impact on cost per patient,^22 27 28^ but may in fact result in increased costs due to extra personnel.^28^ However, since reductions in the number of additional tests required, prescriptions, referrals and admissions have been well documented,^27 29–32^ a beneficial cost-effectiveness of the measure cannot be ruled out.

In this study, we documented a 55% reduction in admissions among patients seen by the GP in comparison to those seen by the ED. However, we observed that a substantial number of patients seen and managed exclusively by the GP were referred to their own GP for further follow-up. This could indicate that universalisation of the measure of incorporating a GP in paediatric ED departments could potentially result in shift of the burden to primary care, and the impact on the whole system requires further investigation.

Finally, the GP was significantly more likely to prescribe antibiotics than the ED, suggesting that the permanent inclusion of a GP within the ED would require a close monitoring of prescription practices to avoid overprescription or inappropriate prescription of antibiotics. Judicious antimicrobial use involves prescribing antibiotics only for probable or definite bacterial infections, rather than for probable viral infections such as upper respiratory tract infections. Standardisation of antibiotic prescription guidelines at a national level, periodic training and continuous monitoring of prescription practices among clinicians with prescribing authority would be beneficial to overturn this trend. A recent study reported that a feedback regarding prescribing can significantly reduce unnecessary antibiotic use.^33^

Our intervention provided evidence that the presence of the GP in a busy paediatric ED resulted in a significant reduction in waiting times for patients seen by the GP. This has important implications for NHS trusts, as breaching the target of resolving at least 95% of the attendances within 4 hours can have negative economic consequences for hospitals.^34^ However, a decrease in overall time spent in ED and waiting times is to be expected due to the additional resources added to the department with the addition of the GP. The findings of this study need to be considered in the context of current efforts to integrate care and to deliver more services in the community.^35^ Ideally, patients triaged as ‘GP appropriate’ should be seen by GPs in the community. Providing GP services colocated with ED may encourage patients to seek secondary care instead of primary care closer to home. Any service redesign needs to balance cost, clinical effectiveness and patient preferences across the whole system, and should be carefully evaluated.

### Strength and limitations

To our knowledge, this pilot study is the first to assess the impact of a GP service within a large paediatric ED in the UK, and is likely to be generalisable to other similar settings. A strength of the study is the completeness of the data obtained from two reliable sources, the GP and the ED databases, which were matched and carefully cross-checked. We have made use of a natural experiment, and routinely collected data, to pragmatically evaluate the impact of an intervention in one of Europe’s largest paediatric EDs.

Our study has a number of limitations. Data collection was limited to that collected in the routine data systems, and we could not evaluate the time between arrival and being seen by a doctor, which is of importance to patients when they visit an ED, and can have an impact on the overall satisfaction with the service. Second, we extracted the information retrospectively from the databases, and relied on ICD-10 (International Classification of Diseases) codes for diagnostic information, and there is thus scope for misclassification. We did not collect data on staff workload, number of staff in the ED or any placebo outcomes. There was a potential for bias to have arisen through differences in the way that the triage was implemented within GP hours versus outside of GP hours. It is possible that the criteria for determining whether or not a patient was designated ‘GP appropriate’ were implemented differently between 14:00 and 22:00 hours, because the designation would not make a difference to the patient’s care if the patient arrived after 22:00 or before 14:00 hours, when the GP was not present anyway.

We were able to adjust for a number of important confounders in our analysis, and the GP and ED groups were broadly similar in terms of age and case mix. One of the assumptions of our analysis is conditional exchangeability of the comparison groups, but it is possible that the groups differ on the basis of unmeasured confounders. It is possible that the groups differ on the basis of factors related to time of attendance, disease severity and age (which was significantly different at baseline). However, our conclusions do not change in sensitivity analyses restricting the time of attendance to daytime, and stratifying by age. If, for example, there were systematic differences in years since qualification between GPs and ED doctors, this might mean that the time taken to see the GP was shorter. We did not collect data on year of qualification of doctors, so we were unable to explore this.

Furthermore, the fact that all patients were a relatively homogeneous group of less complicated cases reduces the risk of confounding by case mix. Finally, we did not set out to evaluate the cost-effectiveness of the incorporation of a GP within the ED, as evidence would have been challenging to collect in such a brief period.

## Conclusion

The results presented in this study highlight both the advantages and challenges that can arise when integrating a GP service within a busy paediatric ED. Integrative approaches are currently being seen as a plausible solution to meet the needs of overstretched healthcare services, and further research is needed to guide an evidence-guided decision. Our pilot intervention could be seen as a ‘natural experiment’ that provides some evidence that locating a GP in ED can reduce waiting times and admissions, but increases antibiotic prescribing.

## References

[1] NHS England. A&E attendances and emergency admissions 2015-16. 2016 https://www.england.nhs.uk/statistics/statistical-work-areas/ae-waiting-times-and-activity/statistical-work-areasae-waiting-times-and-activityweekly-ae-sitreps-2015-16/ (accessed 6 May 2016).

[2] Hendry SJ, Beattie TF, Heaney D Minor illness and injury: factors influencing attendance at a paediatric accident and emergency department. Arch Dis Child 2005;90:629–33. 10.1136/adc.2004.049502 15908631 PMC1720434

[3] Lowy A, Kohler B, Nicholl J Attendance at accident and emergency departments: unnecessary or inappropriate? J Public Health Med 1994;16:134–40. 7946485 10.1093/oxfordjournals.pubmed.a042947

[4] McHale P, Wood S, Hughes K, et al Who uses emergency departments inappropriately and when - a national cross-sectional study using a monitoring data system. BMC Med 2013;11:258 10.1186/1741-7015-11-258 24330758 PMC3886196

[5] Penson R, Coleman P, Mason S, et al Why do patients with minor or moderate conditions that could be managed in other settings attend the emergency department? Emerg Med J 2012;29:487–91. 10.1136/emj.2010.107276 21561984

[6] Prince M, Worth C A study of ’inappropriate' attendances to a paediatric Accident and Emergency Department. J Public Health Med 1992;14:177–82. 1515201

[7] Stewart MC, Savage JM, Scott MJ, et al Primary medical care in a paediatric accident and emergency department. Ulster Med J 1989;58:29–35. 2773168 PMC2448563

[8] Thompson MI, Lasserson D, McCann L, et al Suitability of emergency department attenders to be assessed in primary care: survey of general practitioner agreement in a random sample of triage records analysed in a service evaluation project. BMJ Open 2013;3:e003612 10.1136/bmjopen-2013-003612 PMC3855530 24319279

[9] Agarwal S, Banerjee J, Baker R, et al Potentially avoidable emergency department attendance: interview study of patients' reasons for attendance. Emerg Med J 2012;29:e3 10.1136/emermed-2011-200585 22205782

[10] Benger JR, Jones V Why are we here? A study of patient actions prior to emergency hospital admission. Emerg Med J 2008;25:424–7. 10.1136/emj.2007.050856 18573957

[11] Smith JK, Roth S Paediatric A&E attendances; findings and consequences. Arch Dis Child 2008;93:812–3. 10.1136/adc.2008.143420 18719168

[12] Truman CD, Reutter L Care-giving and care-seeking behaviours of parents who take their children to an emergency department for non-urgent care. Can J Public Health 2002;93:41–6. 11925699 10.1007/BF03404416 PMC6979798

[13] Williams A, O’Rourke P, Keogh S Making choices: why parents present to the emergency department for non-urgent care. Arch Dis Child 2009;94:817–20. 10.1136/adc.2008.149823 19395399

[14] Armon K, Stephenson T, Gabriel V, et al Determining the common medical presenting problems to an accident and emergency department. Arch Dis Child 2001;84:390–2. 11316679 10.1136/adc.84.5.390 PMC1718762

[15] Ogilvie S, Hopgood K, Higginson I, et al Why do parents use the emergency department for minor injury and illness? A cross-sectional questionnaire. JRSM Open 2016;7:2054270415623695 10.1177/2054270415623695 26981256 PMC4780204

[16] Hitti EA, Lewin JJ, Lopez J, et al Improving door-to-antibiotic time in severely septic emergency department patients. J Emerg Med 2012;42:462–9. 10.1016/j.jemermed.2011.05.015 21737222

[17] Medicine TRCoE. Acute and emergency care: prescribing the remedy, 2014.

[18] Boeke AJ, van Randwijck-Jacobze ME, de Lange-Klerk EM, et al Effectiveness of GPs in accident and emergency departments. Br J Gen Pract 2010;60:e378–84. 10.3399/bjgp10X532369 20883612 PMC2944947

[19] Bosmans JE, Boeke AJ, van Randwijck-Jacobze ME, et al Addition of a general practitioner to the accident and emergency department: a cost-effective innovation in emergency care. Emerg Med J 2012;29:192–6. 10.1136/emj.2010.101949 21441265

[20] Carson DC H, Stern R Primary care and emergency departments Primary Care Foundation. 2010 http://www.primarycarefoundation.co.uk/images/PrimaryCareFoundation/Downloading_Reports/Reports_and_Articles/Primary_Care_and_Emergency_Departments/Primary_Care_and_Emergency_Departments_RELEASE.pdf (accessed 21st June 2016).

[21] Khangura JK, Flodgren G, Perera R, et al Primary care professionals providing non-urgent care in hospital emergency departments. Cochrane Database Syst Rev 2012;11:CD002097 10.1002/14651858.CD002097.pub3 23152213 PMC4164956

[22] Ramlakhan S, Mason S, O’Keeffe C, et al Primary care services located with EDs: a review of effectiveness. Emerg Med J 2016;33 10.1136/emermed-2015-204900 27068868

[23] Wang M, Wild S, Hilfiker G, et al Hospital-integrated general practice: a promising way to manage walk-in patients in emergency departments. J Eval Clin Pract 2014;20:20–6. 10.1111/jep.12074 24033413

[24] van Veen M, Steyerberg EW, Ruige M, et al Manchester triage system in paediatric emergency care: prospective observational study. BMJ 2008;337:a1501. 18809587 10.1136/bmj.a1501 PMC2548283

[25] Munro J, Mason S, Nicholl J Effectiveness of measures to reduce emergency department waiting times: a natural experiment. Emerg Med J 2006;23:35–9. 10.1136/emj.2005.023788 16373801 PMC2564124

[26] Department of Health. The NHS plan. A plan for investment, a plan for reform, Cm 4818-1. NorwichThe Stationery Office, 2000.

[27] Dale J, Lang H, Roberts JA, et al Cost effectiveness of treating primary care patients in accident and emergency: a comparison between general practitioners, senior house officers, and registrars. BMJ 1996;312:1340–4. 8646050 10.1136/bmj.312.7042.1340 PMC2351016

[28] Salisbury C, Hollinghurst S, Montgomery A, et al The impact of co-located NHS walk-in centres on emergency departments. Emerg Med J 2007;24:265–9. 10.1136/emj.2006.042507 17384380 PMC2658232

[29] Jiménez S, de la Red G, Miró O, et al [Effect of the incorporation of a general practitioner on emergency department effectiveness]. Med Clin 2005;125:132–7. 10.1157/13076941 15989853

[30] Kool RB, Homberg DJ, Kamphuis HC Towards integration of general practitioner posts and accident and emergency departments: a case study of two integrated emergency posts in the Netherlands. BMC Health Serv Res 2008;8:225 10.1186/1472-6963-8-225 18983657 PMC2614425

[31] Murphy AW, Bury G, Plunkett PK, et al Randomised controlled trial of general practitioner versus usual medical care in an urban accident and emergency department: process, outcome, and comparative cost. BMJ 1996;312:1135–42. 8620132 10.1136/bmj.312.7039.1135 PMC2350641

[32] Ward P, Huddy J, Hargreaves S, et al Primary care in London: an evaluation of general practitioners working in an inner city accident and emergency department. J Accid Emerg Med 1996;13:11–15. 8821216 10.1136/emj.13.1.11 PMC1342597

[33] Hallsworth M, Chadborn T, Sallis A, et al Provision of social norm feedback to high prescribers of antibiotics in general practice: a pragmatic national randomised controlled trial. Lancet 2016;387:1743–52. 10.1016/S0140-6736(16)00215-4 26898856 PMC4842844

[34] The Kings Fund. How is the health and social care sAystem performing? quarterly report. 2013 https://www.kingsfund.org.uk/publications/how-health-and-social-care-system-performing-january-2014

[35] Klaber RE, Blair M, Lemer C, et al Whole population integrated child health: moving beyond pathways. Arch Dis Child 2017;102 10.1136/archdischild-2016-310485 27217582

