# Supplementary material for: To GP or not to GP: a natural experiment in children triaged to see a GP in a tertiary paediatric emergency department (ED)
Source: BMJ Qual Saf. 2017 Sep 29;27(7):521–8. doi: 10.1136/bmjqs-2017-006605 (PMC6047147; doi:10.1136/bmjqs-2017-006605)
Supplement: Supplementary file 1 [file bmjqs-2017-006605supp001.docx]

**Supplementary data**

**Table S1:** Characteristics of the “GP appropriate” patients attending the ED at Alder Hey with standardised differences

| ***Variable*** | **GP hours ED Total**  **2821 (54%) 2402 (46%) 5223** | ***Standardised difference*** |
| --- | --- | --- |
| **Gender**  Female  Male | 1380 (48.9%) 1170 (48.7%) 2550 (48.8%)  1441 (51.1%) 1232 (51.3%) 2673 (51.2%) | 0.004 (-3.94, 3.94)  -0.004 (-3.80, 3.80) |
| **Age categorical**  < 1 yr  1-5 yrs  5-10 yrs  >10 yrs | 761 (27%) 574 (23.9%) 1335 (25.6%)  1244 (44.1%) 1006 (41.9%) 2250 (43.1%)  479 (17.0%) 481 (20.0%) 960 (18.4%)  337 (11.9%) 341 (14.2%) 678 (13.0%) | 0.0712 (-4.65, 4.79)  0.0444 (4.10, 4.14)  -0.0773 (-4.99, 4.53)  -0.0683 (-5.14, 5.00) |
| **Deprivation quintiles**  1  2  3  4  5 | 67 (2.4%) 76 (3.2%) 143 (2.7%)  144 (5.1%) 129 (5.4%) 273 (5.2%)  290 (10.3%) 238 (9.9%) 528 (10.1%)  386 (13.7%) 308 (12.8%) 694 (13.3%)  1933 (68.5%) 1647 (68.6%) 3180 (68.5%) | -0.0485 (-5.47, 5.37)  -0.0135 (-5.63, 5.61)  -0.0133 (-5.15, 5.18)  0.0265 (-5.07, 5.12)  -0.0022 (-3.05, 3.05) |

Table S2: Discharge status by PP analysis

| ***Discharge*** | GP hours GP to ED ED Total |
| --- | --- |
| Own GP follow-up  Discharged  Admitted  Outpatient services  A&E services  Community service  Dentist  Left before being seen | 1132 (49.2%) 15 (11.4%) 288 (16.8%) 1435 (34.6%)  1115 (48.4%) 53 (70.0%) 1076 (62.7%) 2261 (54.5%)  6 (0.3%) 35 (26.5%) 138 (6.0%) 179 (4.3%)  5 (0.2%) 8 (6.1%) 85 (5.0%) 98 (2.4%)  3 (0.1%) 1 (0.8%) 15 (0.9%) 14 (0.5%)  1 (0.1%) 2 (1.5%) 4 (0.2%) 7 (0.2%)  1 (0.8%) 1 (0.1%) 2 (0.1%)  40 (1.7%) 108 (6.3%) 148 (4.3%) |

Table S3: Outcomes by PP analysis

| ***Outcome*** | GP hours ED Odds ratio |
| --- | --- |
| Admitted  Antibiotics  Wait exceeded 4hrs  Left before seen | 41 (1.7%) 138 (8.0%) 0.20 (0.14, 0.28)  647 (27.0%) 464 (17.5%) 1.74 (1.48, 2.03)  40 (1.6%) 108 (6.3%) 0.25 (0.17, 0.36)  36 (1.5%) 85 (5.0%) 0.29 (0.19, 0.43) |

Table S4: Sensitivity analysis of patients seen between 10:00 – 18:00 n=2781 by ITT analysis

| ***Outcome*** | GP hours ED Odds ratio |
| --- | --- |
| Admitted  Antibiotics  Wait exceeded 4hrs  Left before seen | 30 (2.1%) 65 (4.8%) 0.44 (0.28, 0.67)  390 (28.2%) 298 (12.7%) 1.33 (1.12, 1.59)  37 (2.6%) 54 (4.0%) 0.45 (0.33, 0.61)  31 (2.2%) 49 (3.6%) 0.65 (0.43, 0.99) |

Table S5: Sensitivity analysis of patients seen between 10:00 – 18:00 n=2781 by PP analysis

| ***Outcome*** | GP hours ED Odds ratio |
| --- | --- |
| Admitted  Antibiotics  Wait exceeded 4hrs  Left before seen | 26 (2.0%) 47 (6.9%) 0.27 (0.17, 0.44)  377 (28.8%) 116 (17.6%) 1.89 (1.50, 2.39)  29 (2.2%) 14 (2.0%) 1.07 (0.56, 2.03)  28 (2.1%) 26 (3.8%) 0.54 (0.32, 0.93) |

Table S6: Sensitivity analysis of patients seen between 10:00 – 18:00 n=2781 by ITT analysis

excluding patients with diagnosis of “other “

| ***Outcome*** | GP hours ED Odds ratio |
| --- | --- |
| Admitted  Antibiotics  Wait exceeded 4hrs  Left before seen | 48 (1.8%) 60 (2.8%) 0.64 (0.44, 0.94)  690 (26.4%) 435 (21.0%) 1.75 (1.17, 1.54)  64 (2.4%) 116 (5.4%) 0.43 (0.32, 0.59)  72 (2.7%) 103 (4.8%) 0.55 (0.41, 0.75) |

Table S7: Sensitivity analysis of children stratified by age

| ***Outcome*** | <=5 >5 Odds ratio |
| --- | --- |
| Admitted  Antibiotics  Wait exceeded 4hrs  Left before seen | 152 (4.2%) 66 (4.1%) 1.04 (0.77, 1.40)  827 (24.0%) 350 (22.0%) 1.09 (0.95, 1.26)  130 (3.6%) 58 (3.6%) 1.01 (0.74, 1.38)  161 (4.5%) 64 (4.0%) 1.14 (0.85, 1.53) |

Table S8: Sensitivity analysis with data split by first and second half of the month

| ***Outcome*** | GP 1^st^ half GP 2^nd^ half Odds ratio |
| --- | --- |
| Admitted  Antibiotics  Wait exceeded 4hrs  Left before seen | 28 (2.0%) 34 (2.4%) 1.18 (0.71, 1.96)  362 (26.8%) 351 (25.3%) 0.93 (0.78, 1.10)  33 (2.4%) 33 (2.3%) 0.97 (0.59, 1.58)  42 (3.0%) 46 (3.2%) 1.06 (0.70, 1.63) |

Figure S1: Antibiotics prescribed to “GP appropriate” patients according to GP group and ED group by percentage of prescriptions


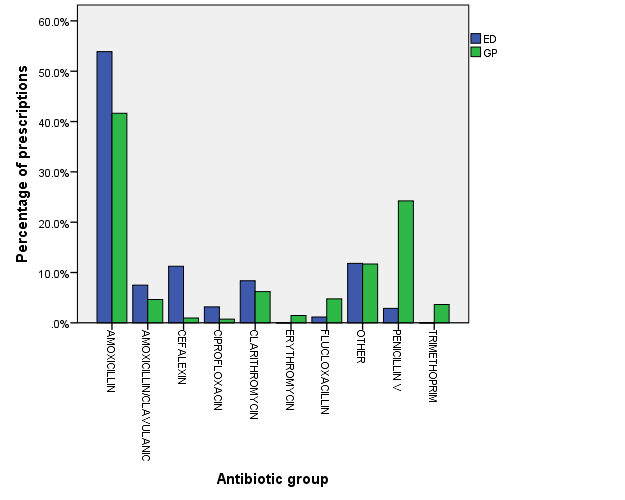


Appendix S9: Assessing how big the unobserved confounding would need to be to change the estimated treatment effects significantly.

The odds for admission comparing children seen in non-GP hours compared to GP hours was 1/0.32 = 3.125 95%CI 2.27 to 4.16. Following Ping 2016, we are interested in assessing if the observed OR of the exposure *E* on the outcome *D* might be completely due to the existence of a common confounder U. For a binary unmeasured confounder to completely explain away the observed OR, both the exposure–confounder OR and the confounder–outcome OR would have to be at least 3.125. Let us now assume then that both the exposure–confounder OR and the confounder–outcome OR have the magnitude 3.125. The joint bounding factor is (3.125*3.125)/(3.125 + 3.125 - 1) = 1.86. Even if we assume such a strong confounder, the point estimate of the causal OR of non-GP hours and admission must still be at least as large as 3.125/1.86 = 1.76 with confidence intervals 1.22 to 2.23. In fact, to explain away the point estimate of the observed OR of 3.125, the magnitude of OR*_EU_* and OR*_UD_* (if OR*_EU_* = OR*_UD_*) should be at least as large as 3.125 + sqrt(3.125 - 1) = 4.58. And to explain away the lower confidence limit 2.27, these two confounding risks should be at least as large as 2.27 + sqrt(2.27 - 1) = 3.4. Since these risks are relatively large, this provides some reassurance that are results are robust to unmeasured confounding.

Ref: Ping D, VanderWeele TJ. Epidemiology. Sensitivity Analysis Without Assumptions 2016 May; 27(3): 368–377.
