# Supplementary figures and images for: To GP or not to GP: a natural experiment in children triaged to see a GP in a tertiary paediatric emergency department (ED)
Source: BMJ Qual Saf. 2017 Sep 29;27(7):521–8. doi: 10.1136/bmjqs-2017-006605 (PMC6047147; doi:10.1136/bmjqs-2017-006605)

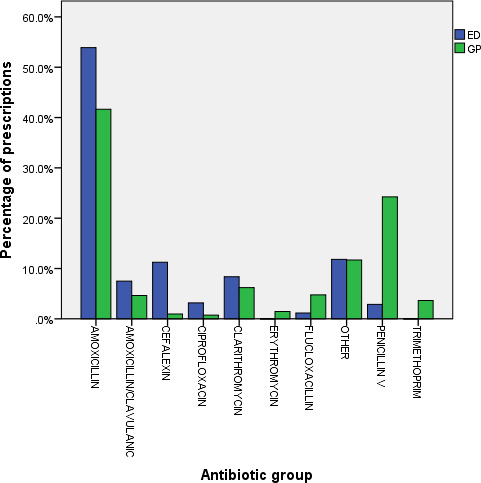

Supplement: Supplementary file 2 [file bmjqs-2017-006605supp002.jpg]
